# Cumulative exposure to metabolic syndrome in a national population-based cohort of young adults and sex-specific risk for type 2 diabetes

**DOI:** 10.1186/s13098-023-01030-z

**Published:** 2023-04-24

**Authors:** Min-Kyung Lee, Jae-Hyuk Lee, Seo Young Sohn, Jiyeon Ahn, Oak-Kee Hong, Mee-Kyoung Kim, Ki-Hyun Baek, Ki-Ho Song, Kyungdo Han, Hyuk-Sang Kwon

**Affiliations:** 1 Division of Endocrinology and Metabolism, Department of Internal Medicine, Myongji Hospital, Hanyang University College of Medicine, Seoul, Gyeonggi-do Republic of Korea; 2 Division of Endocrinology and Metabolism, Department of Internal Medicine, Yeouido St. Mary’s Hospital, College of Medicine, The Catholic University of Korea, 10, 63-ro, Yeongdeungpo-gu, 07345,6 Seoul, Republic of Korea; 3 Department of Statistics and Actuarial Science, Soongsil University, 369 Sangdo-ro, Dongjak- gu, 06978 Seoul, Republic of Korea

**Keywords:** Cumulative burden, Metabolic syndrome, Type 2 diabetes, Young adults

## Abstract

**Background:**

Metabolic syndrome is associated with type 2 diabetes and its prevalence is increasing worldwide in young adults. We aimed to determine whether cumulative exposure to metabolic syndrome is associated with type 2 diabetes risk in young adults.

**Methods:**

Data of 1,376,540 participants aged 20–39 years without a history of type 2 diabetes and who underwent four annual health check-ups were collected. In this large-scale prospective cohort study, we evaluated the incidence rates and hazard ratios (HRs) of diabetes according to cumulative frequencies of metabolic syndrome over 4 years of consecutive annual health check-ups (burden score 0–4). Subgroup analyses were performed by sex and age.

**Results:**

During 5.18 years of follow-up, 18,155 young adults developed type 2 diabetes. The incidence of type 2 diabetes increased with burden score (*P* < 0.0001). The multivariable-adjusted HRs for type 2 diabetes were 4.757, 10.511, 18.288, and 31.749 in participants with a burden score of 1 to 4, respectively, compared to those with 0. In subgroup analyses, the risk of incident diabetes was greater in women than men and in the 20–29 years age group than the 30–39 years age group. The HRs were 47.473 in women and 27.852 in men with four burden scores.

**Conclusion:**

The risk of type 2 diabetes significantly increased with an increase in the cumulative burden of metabolic syndrome in young adults. Additionally, the association between cumulative burden and diabetes risk was stronger in women and the 20s age group.

**Supplementary Information:**

The online version contains supplementary material available at 10.1186/s13098-023-01030-z.

## Background

Metabolic syndrome is a complex disorder defined by a clustering of interrelated medical conditions, including abdominal obesity, high blood pressure, high blood glucose, high serum triglyceride, and low serum high-density lipoprotein (HDL) [1]. Its prevalence is increasing worldwide, and there has been a substantial increase in young adults, driven mostly by the increase in obesity [2]. Obesity, particularly abdominal obesity, is associated with insulin resistance, which leads to hyperglycemia, abnormal lipid profiles, hypertension, and vascular endothelial dysfunction at a younger age [3–6]. Metabolic syndrome is associated with an increased risk of cardiovascular atherosclerotic diseases and type 2 diabetes [7]. Although metabolic syndrome is a strong predictor of incident diabetes, its clinical value for diabetes prediction remains uncertain [8].

Type 2 diabetes in young adults is becoming increasingly prevalent, and early and aggressive risk factor management is warranted [9]. Young-onset type 2 diabetes leads to longer disease exposure and long-term complication over time, resulting in premature mortality and morbidity [10]. Management of type 2 diabetes in this age group is challenging, and there are age-specific concerns [11]. Prospective longitudinal cohort studies have demonstrated that the effect of metabolic syndrome on the risk of type 2 diabetes is greater in young adults than in older adults [12, 13]. Although the absolute rate of incident diabetes is higher in older than in young adults, young adults with metabolic syndrome have a greater risk of incident diabetes than compared to older adults with it. Thus, early assessment of metabolic syndrome and diabetes risk in young adults may provide insights into preventive and control plans for at-risk populations.

The status of metabolic syndrome and its components change over time, and modification of these risk factors may affect various health outcomes, including type 2 diabetes [14]. However, as previous studies have not evaluated the risk for type 2 diabetes according to cumulative exposure to metabolic syndrome in young adults, it would be clinically useful to investigate the relationship between metabolic syndrome and incident diabetes in this age group. In the present large-scale study of a Korean cohort, we aimed to assess the association between cumulative exposure to metabolic syndrome and the risk of incident diabetes in young adults during four annual health check-ups. Moreover, we prospectively evaluated sex- and age-specific diabetes risks according to the cumulative burden of metabolic syndrome.

## Methods

### Data source and study population

This study used the South Korean National Health Insurance Service (NHIS) database, which is a cohort based on nationwide health insurance data [15]. The NHIS includes a stratified random sample for age, sex, eligibility status, region, and income level based on the Korean population. The NHIS conducts annual or biennial health check-ups for all insured Koreans aged ≥ 40 years and for employees aged ≥ 20 years. In this study, we collected special-purpose cohort databases, including local householders and employees aged < 40 years. The database contains health records, including sociodemographic information, anthropometric measurements, laboratory tests, completed questionnaires on lifestyle behaviors, claims for disease diagnosis codes of the International Classification of Diseases, 10th revision [ICD-10], and treatment information for the Korean population.

Of the 6,891,399 young adults aged 20–39 years who underwent health check-ups between 2009 and 2012, we collected data from 1,571,091 participants who completed four consecutive annual health check-ups. We excluded 21,496 participants with missing information for at least one variable and 3,782,119 participants with type 2 diabetes before the index year. Ultimately, the final study population consisted of 1,376,540 participants who had values for all metabolic syndrome components measured. And they were followed to the date of type 2 diabetes diagnosis or until the end of 2018, of which the median follow-up duration is 5.18 years (Supplementary Fig. 1).

### Definition of metabolic syndrome

According to the revised National Cholesterol Education Program Adult Treatment Panel III (NCEP ATP III) criteria [1, 16], metabolic syndrome was diagnosed when three or more of the following five criteria were met: *1*) elevated blood pressure (BP) (≥ 130 mmHg systolic BP, ≥ 85 mmHg diastolic BP, on antihypertensive drug treatment at baseline, and/or a history of hypertension); *2*) elevated triglycerides (fasting triglycerides ≥ 150 mg/dL) or on drug treatment for elevated triglycerides at baseline; *(3)* reduced HDL cholesterol (< 40 mg/dL for men and < 50 mg/dL for women); *(4)* elevated fasting plasma glucose (FPG) level (≥ 100 mg/dL or on drug treatment for elevated glucose), and *(5)* abdominal obesity (waist circumference [WC] ≥ 90 cm for men or 85 cm for women) [17]. Impaired fasting glucose (IFG) was defined as a FPG level of 100–125 mg/dL, except in participants with type 2 diabetes [18].

### Scoring of cumulative burden to metabolic syndrome

To estimate the cumulative effect of exposure to metabolic syndrome, we counted the frequency of metabolic syndrome diagnoses during four years of consecutive annual health check-ups and defined it as the metabolic syndrome burden score. Although metabolic syndrome components are commonly used for metabolic syndrome burden measure, we defined the metabolic syndrome burden as cumulative exposure to metabolic syndrome diagnosis over time to evaluate the association of exposure duration with type 2 diabetes. In this classification, a score of 0 indicated no diagnosis of metabolic syndrome, and scores of 1–4 indicated the number of metabolic syndrome diagnoses over four years. The participants were categorized into five groups according to the burden scores of metabolic syndrome (0, 1, 2, 3, and 4).

### Outcome and follow-up

For each participant, the primary outcome of this study was incident type 2 diabetes, defined as follows [19]: (1) claims with ICD-10 codes E11–E14 and at least one prescription of an antidiabetic medication, or (2) a fasting plasma glucose level ≥ 126 mg/dL (≥ 7.0 mmol/L) from the NHIS health check-up. The study population was followed up from baseline to the date of diagnosis or until the end of the study (between January 1, 2013 and December 31, 2018), and the number of person-years of follow-up was determined.

### Measurements and definitions of variables

All participants were required to complete standardized self-administered questionnaires that inquired about smoking status, alcohol consumption, physical activity, yearly income, and medical history. Participants were divided into current and noncurrent smokers. Heavy alcohol consumption was defined as a consumption of ≥ 30 g/day. Regular exercise was defined as moderate-to-high-intensity activity ≥ 3 times/week. The low-income level was dichotomized as the lowest 25%. Physical examination was performed by measuring height, weight, WC, systolic BP, and diastolic BP, according to standardized methods. Body mass index (BMI) was calculated as the weight in kilograms divided by the height in square meters (kg/m^2^). Blood samples for the measurement of FPG, triglyceride, and HDL cholesterol levels were obtained in the morning after an overnight fast. Comorbidities were defined using ICD-10 diagnosis codes, prescription information from the year prior to the assessment, and health check-up results. Diagnoses of hypertension and dyslipidemia were defined using laboratory and anthropometric measurement data (systolic BP ≥ 140 mmHg or diastolic BP ≥ 90 mmHg; total cholesterol levels ≥ 240 mg/dl), ICD codes (ICD I10–I13 or I15; E78), and medication use, including antihypertensive or dyslipidemia medication. The hospitals where these health check-ups were performed were certified by the NHIS and subjected to regular quality control.

### Statistical analysis

The baseline characteristics of the study population are presented as mean ± standard deviation (SD) or proportions (%). Geometric means were used for heavily skewed distributions. The 95% confidence intervals (CI) were calculated using the Wald method for means. The incidence rates of diabetes were calculated by dividing the number of incident cases by the total follow-up period and were presented per 1,000 person-years. The disease-free probability of type 2 diabetes was calculated using Kaplan–Meier curves, and a log-rank test was performed to analyze the differences by metabolic syndrome burden scores. Cox proportional hazards regression models were used to estimate hazard ratios (HRs) and 95% CIs for incident diabetes by adjusting for important risk factors such as sex, age, smoking status, alcohol consumption, and physical activity. Stratified analyses were performed by sex (men vs. women) and age (20–29 vs. 30–39 years), and the interactions between subgroups were tested. In addition, we conducted a stratified analysis to evaluate whether the cumulative burden of metabolic syndrome was consistently associated with an increased risk of type 2 diabetes and assessed the P-value for interactions. All statistical tests were two-sided, and statistical significance was set at P ≤ 0.05. All analyses were performed using the Statistical Analysis System statistical software package (version 9.4; SAS Institute Inc., Cary, NC, USA).

## Results

### Baseline characteristics of the study population

The baseline characteristics of the study population, stratified according to the burden score of metabolic syndrome during the four annual health check-ups, are shown in Table 1. Of a total of 1,376,540 participants (984,497 men and 392,043 women), 1,064,241 (77.3%) did not have a diagnosis of metabolic syndrome over four years. A total of 160,944 (11.7%) participants had a one-time diagnosis of metabolic syndrome, 76,096 (5.5%) had two diagnoses, 45,560 (3.3%) had three diagnoses, and 29,699 (2.2%) had four consecutive diagnoses. The mean ages of the study population with burden scores of 0–4 were 32.01, 33.47, 33.87, 34.14, and 34.48 years, respectively. Participants who are current smokers and heavy alcohol drinkers with a low level of income tend to have higher burden scores. BMI, WC, BP, FPG, and triglyceride levels gradually increased, and HDL cholesterol levels decreased as the burden score increased.

**Table 1:** Baseline characteristics of the young adult population according to metabolic syndrome burden scores during the four years

Characteristic	Total	Number of metabolic syndrome diagnosis					*P* for trend
		0	1	2	3	4	
Participants, n	1,376,540	1,064,241	160,944	76,096	45,560	29,699	< 0.0001
Men	984,497(71.52)	697,200(65.51)	145,149(90.19)	70,908(93.18)	42,936(94.24)	28,304(95.3)	
Women	392,043(28.48)	367,041(34.49)	15,795(9.81)	5188(6.82)	2624(5.76)	1395(4.7)	
Age, years	32.41 ± 4.11	32.01 ± 4.16	33.47 ± 3.71	33.87 ± 3.55	34.14 ± 3.46	34.48 ± 3.34	< 0.0001
20–29	352,088(25.58)	311,721(29.29)	24,434(15.18)	8967(11.78)	4600(10.1)	2366(7.97)	
30–39	1,024,452(74.42)	752,520(70.71)	136,510(84.82)	67,129(88.22)	40,960(89.9)	27,333(92.03)	
Current smokers	512,392(37.22)	349,299(32.82)	79,982(49.7)	40,418(53.11)	25,354(55.65)	17,339(58.38)	< 0.0001
Heavy alcohol drinker	126,604(9.2)	81,771(7.68)	21,439(13.32)	11,325(14.88)	7188(15.78)	4881(16.43)	< 0.0001
Regular exercise (yes)	242,926(17.65)	185,673(17.45)	29,865(18.56)	13,983(18.38)	8275(18.16)	5130(17.27)	< 0.0001
Low income (lower 25%)	377,051(27.39)	282,026(26.5)	47,400(29.45)	23,456(30.82)	14,460(31.74)	9709(32.69)	< 0.0001
Body mass index, kg/m²	23.52 ± 3.6	22.53 ± 2.97	25.73 ± 3.16	27.25 ± 3.28	28.47 ± 3.4	29.81 ± 3.55	< 0.0001
Waist circumference, cm	73.68 ± 7.12	70,202(6.6)	52,771(32.79)	39,519(51.93)	30,594(67.15)	24,307(81.84)	< 0.0001
Systolic blood pressure, mmHg	118.91 ± 12.71	116.44 ± 11.69	124.91 ± 11.78	128.01 ± 11.99	130.47 ± 12.49	133.64 ± 13.33	< 0.0001
Diastolic blood pressure, mmHg	74.81 ± 9.14	73.18 ± 8.43	78.67 ± 8.65	80.87 ± 8.98	82.62 ± 9.55	84.99 ± 10.31	< 0.0001
Fasting plasma glucose, mmol/l	90.56 ± 10.25	89.06 ± 9.5	94.22 ± 10.66	96.02 ± 10.94	97.64 ± 11.25	99.32 ± 11.65	< 0.0001
Total cholesterol, mg/dL	189.04 ± 33.86	184.9 ± 31.98	199.79 ± 35.3	205 ± 36.16	207.94 ± 36.9	209.33 ± 38.23	< 0.0001
Triglycerides, mg/dL^*^	104.68(104.57-104.78)	89.7(89.61–89.79)	154.91(154.51-155.31)	186.4(185.73-187.08)	211.72(210.76-212.68)	244.9(243.57-246.23)	< 0.0001
HDL cholesterol, mg/dL	56.21 ± 18.46	58.79 ± 18.42	49.41 ± 16.27	46.57 ± 15.02	44.72 ± 13.86	42.81 ± 14.49	< 0.0001
LDL cholesterol, mg/dL	108.73 ± 32.32	106.01 ± 30.47	116.91 ± 34.94	119.16 ± 36.51	119.74 ± 38.75	117.96 ± 40.32	< 0.0001
Hypertension (%)	99,625(7.24)	37,842(3.56)	20,293(12.61)	15,324(20.14)	13,103(28.76)	13,063(43.98)	< 0.0001
Dyslipidemia (%)	116,293(8.45)	58,991(5.54)	22,972(14.27)	14,568(19.14)	10,641(23.36)	9121(30.71)	< 0.0001
Metabolic syndrome components							
Elevated fasting glucose (%)	239,081(17.37)	125,235(11.77)	49,620(30.83)	29,097(38.24)	20,168(44.27)	14,961(50.38)	< 0.0001
Elevated triglycerides (%)	377,870(27.45)	171,008(16.07)	88,799(55.17)	53,764(70.65)	37,029(81.28)	27,270(91.82)	< 0.0001
Reduced HDL cholesterol (%)	187,631(13.63)	84,550(7.94)	39,935(24.81)	26,348(34.62)	19,848(43.56)	16,950(57.07)	< 0.0001
Elevated blood pressure (%)	392,067(28.48)	209,796(19.71)	78,815(48.97)	46,818(61.52)	32,173(70.62)	24,465(82.38)	< 0.0001
Abdominal obesity (%)	217,393(15.79)	70,202(6.6)	52,771(32.79)	39,519(51.93)	30,594(67.15)	24,307(81.84)	< 0.0001
Follow-up duration, median (years)	5.18 (4.14–5.51)	5.18 (4.13–5.51)	5.18 (4.21–5.5)	5.16 (4.18–5.48)	5.15 (4.12–5.47)	5.12 (3.9–5.44)	< 0.0001

Data are presented as mean ± standard deviation (SD) or proportions (%)

^*^Geometric means

### Cumulative burden of metabolic syndrome and the risk of incident diabetes

**Fig. 1:**
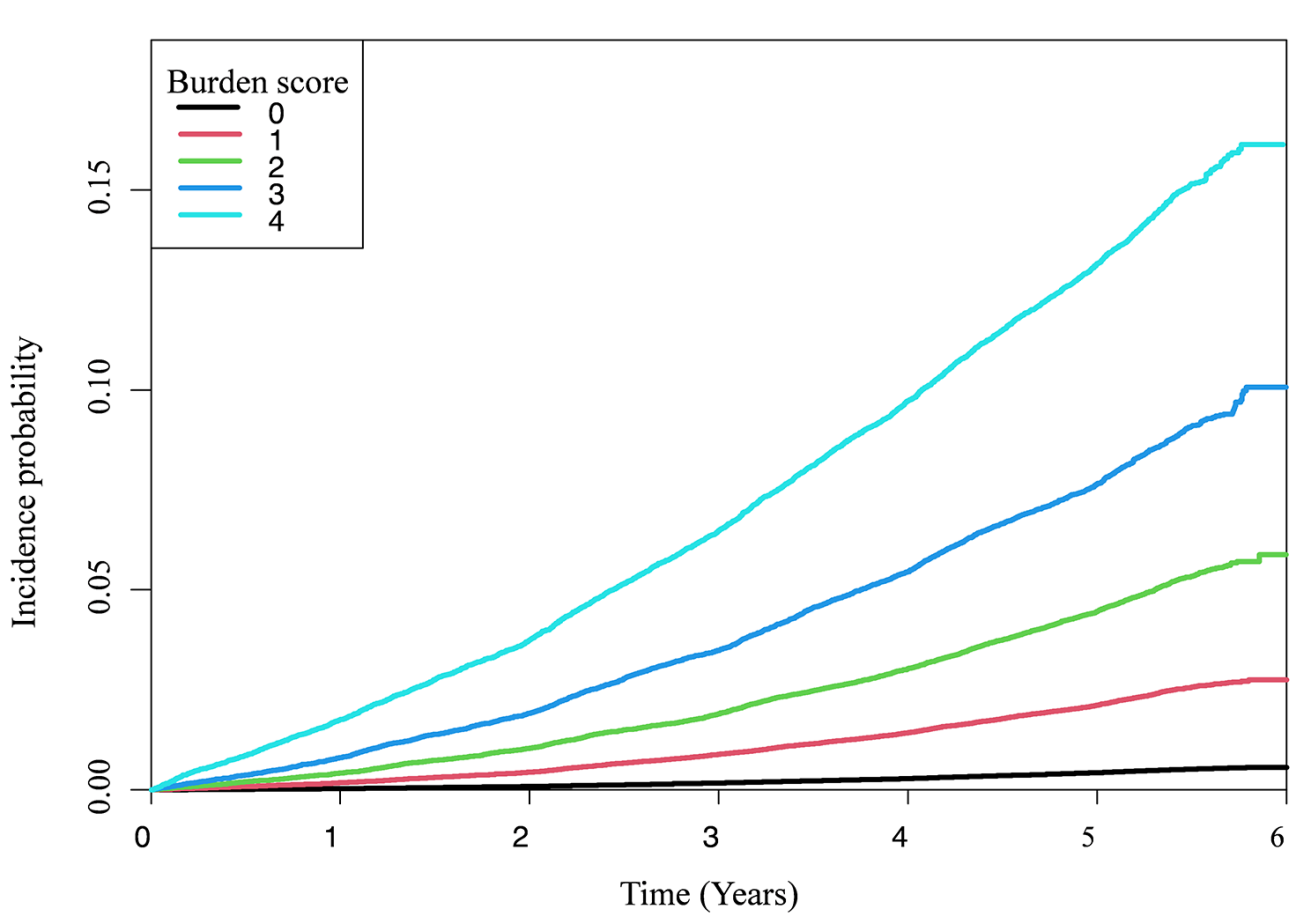
Cumulative incidence of type 2 diabetes according to metabolic syndrome burden scores in young adults

During the median follow-up of 5.18 years (interquartile range, 4.13–5.51 years), 18,155 participants newly developed type 2 diabetes. Table 2 shows the incidence rates and HRs of type 2 diabetes according to the burden scores of metabolic syndrome and its components. The age- and sex-adjusted incidence rates of type 2 diabetes were 0.873, 4.332, 9.188, 16.065, and 27.994 per 1,000 person-years for participants with 0–4 burden scores, respectively. As shown in Fig. 1, the cumulative incidence of type 2 diabetes was significantly different according to metabolic syndrome burden scores (log-rank test, *P* < 0.001). After adjusting for sex, age, smoking status, alcohol consumption, and physical activity, the multivariable-adjusted HRs for incident diabetes were 4.972 (95% CI, 4.745–5.211), 10.511 (95% CI, 10.024–11.022), 18.288 (95% CI, 17.442–19.175), and 31.749 (95% CI, 30.305–33.263) for participants with scores of 1–4, respectively, compared to those with a metabolic syndrome burden score of 0 (Table 2). Additionally, the incidence rate and HRs of type 2 diabetes increased significantly as the burden scores of each metabolic syndrome component increased. The multivariable-adjusted HRs for incident diabetes in participants with four (1–4) scores were 28.223 (95% CI, 26.824–29.694) for elevated FPG, 11.852 (95% CI, 11.423–12.297) for abdominal obesity, 10.807 (95% CI, 10.305–11.333) for elevated triglycerides, 4.828 (95% CI, 4.588–5.082) for reduced HDL cholesterol, and 6.398 (95% CI, 6.098–6.713) for elevated BP, compared with those with a score of 0 (Table 2).

**Table 2:** Multivariable-adjusted hazard ratios for developing type 2 diabetes mellitus according to burden scores of metabolic syndrome and its components in young adults

Diagnosis	Score	Number	Cases, n	Follow-up duration (person-years)	Incidence rate (per 1,000 person-years)	HR (95% CI)		
						Model 1	Model 2	Model 3
Metabolic syndrome	0	1,064,241	4376	5011133.53	0.8733	1(Ref.)	1(Ref.)	1(Ref.)
	1	160,944	3306	763201.11	4.3318	4.944(4.726,5.173)	5.022(4.792,5.262)	4.972(4.745,5.211)
	2	76,096	3293	358396.54	9.1881	10.515(10.05,11.002)	10.652(10.159,11.169)	10.511(10.024,11.022)
	3	45,560	3407	212079.85	16.0647	18.442(17.634,19.287)	18.603(17.744,19.503)	18.288(17.442,19.175)
	4	29,699	3773	134777.37	27.9943	32.372(30.993,33.813)	32.441(30.97,33.981)	31.749(30.305,33.263)
Elevated fasting glucose	0	814,205	3309	3828987.43	0.8642	1(Ref.)	1(Ref.)	1(Ref.)
	1	332,181	3910	1572459.21	2.4866	2.869(2.74,3.005)	2.728(2.603,2.858)	2.698(2.575,2.826)
	2	142,837	4054	674341.6	6.0118	6.943(6.632,7.269)	6.384(6.093,6.689)	6.303(6.015,6.604)
	3	62,869	3835	293176.13	13.0809	15.153(14.465,15.875)	13.605(12.972,14.27)	13.464(12.836,14.123)
	4	24,448	3047	110624.03	27.5437	32.178(30.633,33.801)	28.298(26.898,29.771)	28.223(26.824,29.694)
Abdominal obesity	0	1,052,860	6047	4979558.84	1.2144	1(Ref.)	1(Ref.)	1(Ref.)
	1	118,690	2102	557438.85	3.7708	3.117(2.966,3.276)	2.968(2.824,3.12)	2.962(2.819,3.114)
	2	66,501	1828	309106.22	5.9138	4.911(4.661,5.175)	4.553(4.319,4.8)	4.523(4.291,4.768)
	3	56,865	2260	262475.5	8.6103	7.172(6.834,7.527)	6.576(6.263,6.906)	6.513(6.202,6.839)
	4	81,624	5918	371008.99	15.9511	13.348(12.878,13.836)	12.082(11.645,12.535)	11.852(11.423,12.297)
Elevated triglycerides	0	744,002	3223	3474839.78	0.9275	1(Ref.)	1(Ref.)	1(Ref.)
	1	241,374	2581	1142530.75	2.259	2.421(2.298,2.549)	2.423(2.298,2.555)	2.402(2.278,2.532)
	2	147,727	2680	702536.24	3.8147	4.078(3.875,4.293)	4.067(3.854,4.292)	4.004(3.794,4.226)
	3	118,381	3273	564032.95	5.8029	6.196(5.902,6.505)	6.126(5.814,6.454)	5.997(5.691,6.319)
	4	125,056	6398	595648.69	10.7412	11.457(10.982,11.952)	11.143(10.63,11.681)	10.807(10.305,11.333)
Reduced HDL cholesterol	0	986,960	8553	4634608.81	1.84546	1(Ref.)	1(Ref.)	1(Ref.)
	1	196,903	3537	932971.36	3.79111	2.045(1.967,2.127)	2.044(1.966,2.126)	2.035(1.956,2.116)
	2	93,124	2377	441023.6	5.38973	2.906(2.777,3.041)	2.883(2.755,3.017)	2.873(2.745,3.007)
	3	58,350	1885	276531.41	6.81659	3.671(3.492,3.858)	3.6(3.424,3.784)	3.584(3.409,3.768)
	4	41,203	1803	194453.22	9.27215	4.995(4.747,5.255)	4.847(4.605,5.1)	4.828(4.588,5.082)
Elevated blood pressure	0	626,411	3465	2931444.39	1.18201	1(Ref.)	1(Ref.)	1(Ref.)
	1	303,329	3273	1431605.16	2.28624	1.927(1.837,2.021)	1.815(1.728,1.907)	1.802(1.715,1.893)
	2	198,518	3396	939564.42	3.61444	3.042(2.901,3.19)	2.798(2.661,2.941)	2.762(2.627,2.904)
	3	140,181	3488	664791.39	5.24676	4.411(4.209,4.624)	3.98(3.785,4.185)	3.92(3.728,4.122)
	4	108,101	4533	512183.05	8.85035	7.437(7.116,7.774)	6.478(6.175,6.796)	6.398(6.098,6.713)

Model 1 was crude. Model 2 was adjusted for sex and age. Model 3 was adjusted for sex, age, smoking status, alcohol consumption, and physical activity

### Sex- and age-specific diabetes risk according to metabolic syndrome and IFG burden scores

**Fig. 2:**
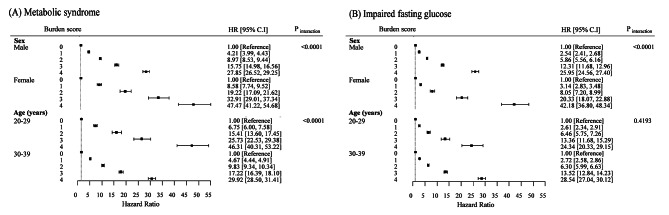
Forest plot showing sex- and age-specific diabetes risk according to burden scores of metabolic syndrome (A) and impaired fasting glucose (B) in young adults

The associations of the burden scores of metabolic syndrome and IFG with diabetes risk by sex (men vs. women) and age (20–29 vs. 30–39 years) are shown in Fig. 2. The forest plot shows that the significant association and increasing trends were consistent regardless of subgroup. The multivariable-adjusted HRs for incident diabetes in participants with metabolic syndrome burden scores of 4 were greater in women (HR: 47.473 [95% CI, 41.216–54.681]) than in men (HR: 27.852 [95% CI, 26.523–29.248]), and in the 20–29 years age group (HR: 46.314 [95% CI, 40.307–53.215]) than in the 30–39 years age group (HR: 29.916 [95% CI, 28.495–31.409]). The interaction between metabolic syndrome burden scores and subgroups (sex and age) was statistically significant (*P* < 0.0001). However, the HR of incident diabetes according to IFG burden scores was not significantly different between the 20–29 and 30–39 years age group (P = 0.4193). In auxiliary analyses, the HR for incident diabetes according to burden scores of abdominal obesity was significantly higher in women than in men (data not shown).

## Discussion

In this large-scale prospective cohort study, we investigated the association between cumulative exposure to metabolic syndrome and diabetes risk in young adults who received four consecutive annual health check-ups. During the 5-year follow-up period, the risk of developing type 2 diabetes increased with the burden scores of metabolic syndrome and its components in young adults in a steep dose-response manner. Additionally, we observed that the risk of diabetes was more strongly associated with the cumulative burden of metabolic syndrome in women and youth in their 20s.

Metabolic syndrome is associated with resistance to the effects of insulin on peripheral glucose and fatty acid utilization, leading to developing type 2 diabetes [20]. Its prevalence is increasing in young adults [21]. Young adults with metabolic syndrome are concerned about the potential for future development of chronic metabolic diseases [22]. Moreover, early detection of young adults at a future risk for type 2 diabetes is important, as the progression to diabetes becomes irreversible after a certain stage [23]. The present study showed that cumulative numbers of diagnosis for metabolic syndrome over the years in young adults might provide a clinical value for predicting the development of type 2 diabetes. Clinical trials have shown that lifestyle modifications focusing on weight reduction and increased physical activity can reduce the development of metabolic syndrome [24]. Thus, we suggest that the key clinical implication of diagnosing metabolic syndrome in young adults may be the identification of a patient who needs aggressive lifestyle modifications to reduce the cumulative burden of metabolic syndrome.

In the exploratory subgroup analyses, we assessed the risk of type 2 diabetes according to the cumulative burden of metabolic syndrome and IFG by sex and age. We found that women had a much stronger effect of cumulative exposure to metabolic syndrome or IFG on diabetes risk than men, suggesting there may be a difference in the burden scores and diabetes risk by sex. Although the mechanism of sex-specific differences in diabetes risk is uncertain, several previous studies have proposed that visceral fat accumulation in women increases susceptibility to type 2 diabetes compared with that in men [25, 26]. Visceral fat is a major source of circulating free fatty acids and cytokines, which directly induce insulin resistance and atherogenic lipid profile [27]. Sex differences in visceral fat accumulation may contribute to sex differences in type 2 diabetes. Studies have consistently reported a more prominent association between obesity exposure and diabetes risk in young women [28, 29]. The supplementary results of our study demonstrated that young women had a stronger positive association between cumulative exposure to abdominal obesity and incident diabetes, which is consistent with the findings of previous studies. On this basis, it could be proposed that the greater the exposure to abdominal obesity in young women, the higher the risk of type 2 diabetes. The cause of this difference is unclear; sex hormones are considered to play an important role in the relationship between fat distribution and metabolic health in men and women [30]. Further studies are needed to clarify the sex-specific mechanisms of differences in diabetes risk related to abdominal obesity, metabolic syndrome, or IFG.

Previous meta-analyses have shown that the cardiovascular disease risk conferred by metabolic syndrome is higher in women than in men [31]. Several unique features of metabolic syndrome, such as an insulin-resistant state and increased abdominal fat, may be associated with sex differences in the risk of type 2 diabetes and cardiovascular disease [32]. The current research suggests that the pathophysiology of metabolic syndrome and its contribution to the relative risk of cardiovascular disease and type 2 diabetes show sex differences, which might be of potential relevance for prevention, diagnostics, and therapy of metabolic syndrome. In recent years, the prevalence of metabolic syndrome has increased, and this increase has been steeper in women aged 20–39 years [33]. Therefore, sex-specific strategies for the management of metabolic risk factors to prevent type 2 diabetes should be developed in young adults.

While metabolic syndrome predicts an increased risk for diabetes, it is not clear whether the condition is superior to FPG in identifying individuals at a high risk of developing diabetes. In a prospective cohort study of 19,475,643 Korean adults, IFG was particularly superior in predicting incident diabetes among the five components of metabolic syndrome [34]. In the present study, we also examined the risk of incident diabetes cumulative exposure to each component of metabolic syndrome over the 4 years. We observed that the cumulative burden of all components was significantly associated with incident diabetes, and persistent exposure to elevated FPG levels was the most robust individual predictor of type 2 diabetes. Cultural, educational, and socioeconomic phenomena may influence the prevalence of the diagnostic components of metabolic syndrome through differences in lifestyle [35]. Our findings were determined after adjusting for age, sex, alcohol consumption, smoking status, and physical activity.

In addition, the present study showed that cumulative exposure to metabolic syndrome was associated with a greater risk of incident diabetes than was elevated FPG. A recent study showed that cumulative exposure to IFG was associated with a higher risk of type 2 diabetes [36]. Some studies have reported that metabolic syndrome is a more potent risk factor for developing diabetes than elevated FPG levels [37]. Another study reported conflicting results that metabolic syndrome was not superior to the measurement of blood glucose alone in predicting diabetes [38]. A thorough assessment of whether metabolic syndrome is an improvement in the prediction of incident diabetes in young adults over the simple measurement of fasting glucose is lacking. However, based on our findings, the cumulative burden of IFG could not be the best and most practical predictor of incident diabetes in the 20s youth group. Therefore, we suggest that persistent exposure to metabolic syndrome may be a more specific predictor of diabetes risk in young adults.

In the present study, the risk of incident diabetes was higher in the 20–29 years age group than in the 30–39 years age group across the cumulative burden of metabolic syndrome. However, there was no significant difference in diabetes risk according to the cumulative burden of IFG between the groups. We found that in youth in their 20s, the cumulative burden of metabolic syndrome showed a higher association with incident diabetes than that of IFG. Therefore, early detection of metabolic syndrome is vital, especially in the 20s youth group; lifestyle intervention and possibly pharmacotherapy, if its safety has been clearly demonstrated, should be followed to minimize the global socioeconomic burden of type 2 diabetes.

The strengths of our study included its longitudinal population-based design, sufficient number of type 2 diabetes events, high follow-up rate, and nationally representative data derived from the entire Korean population. However, the present study had some limitations that should be considered. First, the diagnosis of metabolic syndrome was done through single-time annual health check-ups. However, this method is widely used in other epidemiological studies and we complement this limitation by examining cumulative exposure to metabolic syndrome from consecutive examinations over four years. Therefore, this study assessed repetitive exposure to metabolic syndrome during general health screening. Second, because type 2 diabetes was defined based on the prescription of antidiabetic medication, the presence of relevant ICD-10 codes, and FPG levels from a health check-up, the incidence of type 2 diabetes might have been underestimated. Third, since some of the participants did not engage in the health screening program or receive long-term care services, there was a possibility of selection bias in the health screening information. Lastly, variables on family history, genetic predisposition, and health behaviors were limited since those data were obtained from self-reporting questionnaires in nationwide health screenings.

## Conclusion

Cumulative exposure to metabolic syndrome and its components in young adults was associated with an increased risk of developing type 2 diabetes in a steep dose-response manner. Furthermore, the cumulative burden of metabolic syndrome had a much greater impact on incident diabetes than cumulative exposure to IFG in young adults. In subgroup analyses by sex and age, the association between the cumulative burden of metabolic syndrome and diabetes risk was strong in women and youth in their 20s. Although the current study could not establish causal relationships, reducing cumulative exposure to metabolic syndrome may provide new strategies for preventing type 2 diabetes in women in their 20s.

## Electronic supplementary material

Below is the link to the electronic supplementary material.

Supplementary Material 1

## Data Availability

The authors are restricted from sharing the data underlying this study because The Korean National Health Insurance Service (NHIS) owns the data. Researchers can request access on the NHIS website (https://nhiss.nhis.or.kr). Details of this process and a provision guide are now available at http://nhiss.nhis.or.kr/bd/ab/bdaba000eng.do.

## List of abbreviations

NHIS: National Health Insurance Service
NCEP ATP III: National Cholesterol Education Program Adult Treatment Panel III
HDL: High density lipoprotein
BP: Blood pressure
FPG: Fasting plasma glucose
WC: Waist circumference
IFG: Impaired fasting glucose
BMI: Body mass index:SD:Standard deviation
CI: Confidence intervals
HR: Hazard ratio
