# Supplementary material for: Cumulative exposure to metabolic syndrome in a national population-based cohort of young adults and sex-specific risk for type 2 diabetes
Source: Diabetol Metab Syndr. 2023 Apr 24;15:78. doi: 10.1186/s13098-023-01030-z (PMC10123975; doi:10.1186/s13098-023-01030-z)
Supplement: Supplementary file 1 — Supplementary Material 1 [file 13098_2023_1030_MOESM1_ESM.pdf]

Supplementary figure 1. Flow diagram of the study population

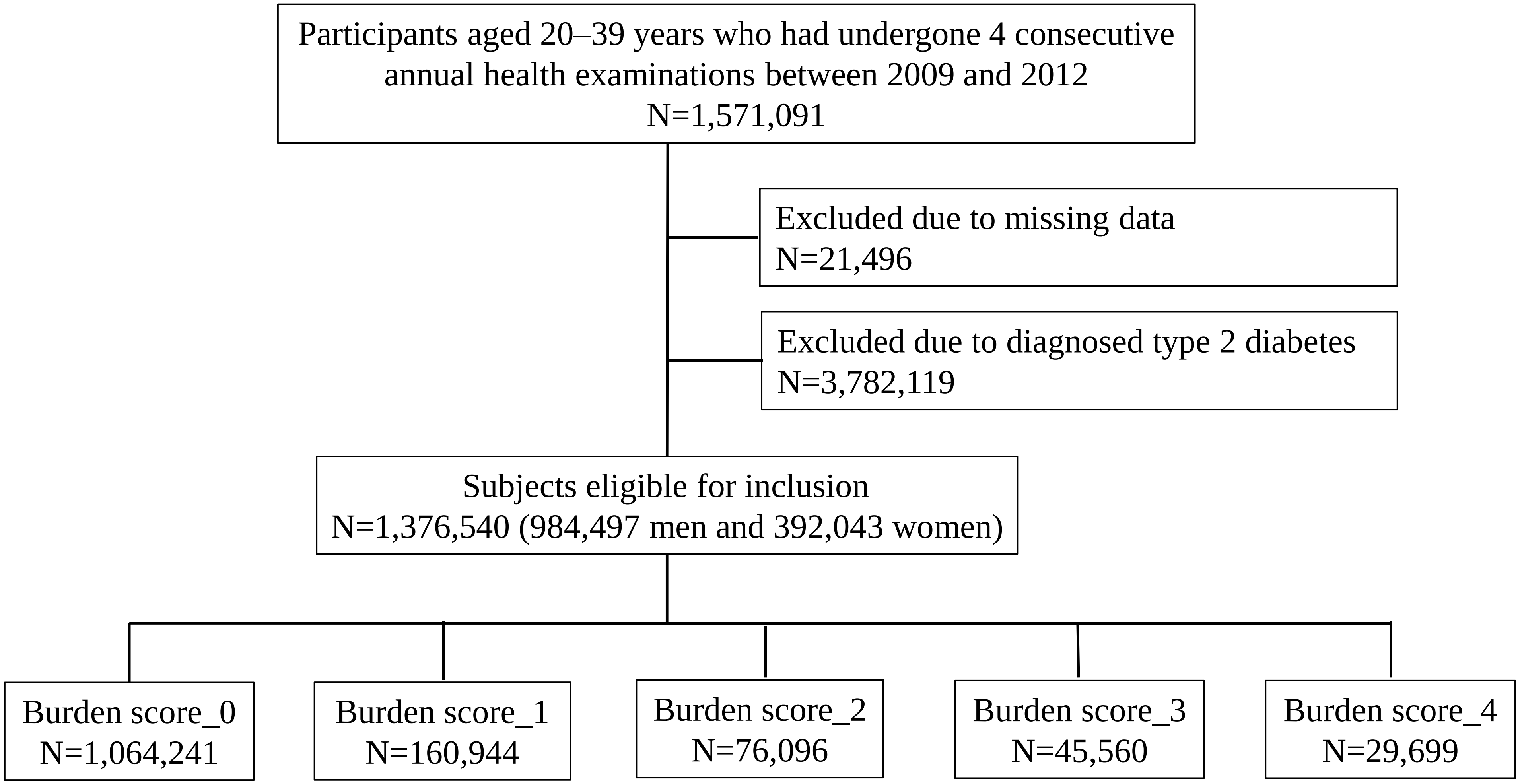

Followed to the date of type 2 diabetes diagnosis or until the end of 2018

(Median follow-up duration: 5.18 years)
